# Investing in Health Innovation: A Cornerstone to Achieving Global Health Convergence

**DOI:** 10.1371/journal.pbio.1002389

**Published:** 2016-03-02

**Authors:** Gavin Yamey, Carlos Morel

**Affiliations:** 1 Duke Global Health Institute, Duke University, Durham, North Carolina, United States of America; 2 National Institute of Science and Technology for Innovation in Neglected Diseases, Centre for Technological Development in Health, Oswaldo Cruz Foundation, Rio de Janeiro, Brazil

## Abstract

Gavin Yamey and Carlos Morel, coordinators of a new PLOS collection on the innovations needed to reduce infectious, maternal, and child deaths to universally low levels, highlight the key cross-cutting themes emerging from the collection.

## Introduction

In December 2013, the Commission on Investing in Health—a group of 25 economists and global health experts chaired by former United States Treasury Secretary Lawrence Summers and health economist Dean Jamison—published its report, called “Global Health 2035” [1]. The report set out an ambitious investment framework for achieving what the authors called a “grand convergence in global health” by 2035. They defined grand convergence as a reduction in avertable infectious, maternal, and child deaths to universally low levels—the kind of levels seen today in the world’s best-performing middle-income countries, such as Chile, China, Costa Rica, and Cuba (conveniently labeled “the 4C countries”). Modeling by the Commission suggested that convergence within a generation could be achieved by aggressively scaling up health tools (e.g., medicines, vaccines, and diagnostics) and strengthening health systems to deliver these tools. But the report came to an important conclusion: The world cannot reach convergence with today’s tools alone. Tomorrow’s tools will also be needed.

The Commission researchers first modeled the health impact of building robust health delivery systems and scaling up today’s medicines, vaccines, diagnostic tests, and other health technologies to extremely high coverage levels, such that around 90%–95% of those who need them are receiving them [1]. Even under these optimistic conditions, the modeling showed that low-income countries would reach about two-thirds of the way to convergence by 2035 [1]. The remaining gap could be closed only through the discovery, development, and delivery of new health technologies. Empirical research has shown that countries that adopt such innovations see acceleration in their health progress [1,2]. These “rapid adopters” achieve an additional decrease in their under-five mortality rate of about 2% per year, compared with countries that do not take up these technologies [2]. In the second step of the Commission’s modeling, the impact of new technologies was included—i.e., an additional 2% per year decline in mortality rates was applied. Only with this “accelerator” effect was convergence achieved.

What are the most important innovation priorities for closing the convergence gap? Which tools in the pipeline appear to have the most potential as “game changers”? These questions are at the heart of a special collection of nine articles, called “Grand Convergence: Aligning Technologies and Realities in Global Health,” being published today across three PLOS journals: *PLOS Biology*, *PLOS Medicine*, and *PLOS Neglected Tropical Diseases*. Over the last 18 months, we have had the privilege of acting as Collection Coordinators, choosing the topics and authors and shaping the overall collection structure (we played no role in mediating the peer review or in editorial decision-making—as always, these were the sole responsibility of the editors of the three journals).

In commissioning the collection, we aimed to focus on five conditions that disproportionately affect the world’s poorest people: HIV/AIDS, tuberculosis (TB), malaria, maternal and child mortality, and neglected tropical diseases. While noncommunicable diseases are not a focus of this collection, the take-home messages of the collection on the importance of investing in innovation apply equally to these diseases. We reached out to a diverse group of authors from low-, middle-, and high-income countries, many of whom are directing global disease control campaigns or major international research efforts. Many of the articles in the collection are analyses of potential “game-changing” technologies. We also included provocative pieces that lay out compelling ideas for the kinds of innovation that will be needed in the scale-up and delivery of health tools and services and in the very ways in which we fund and organize health research and development (R&D) worldwide. When we consider all of the articles as a whole, we see three dominant cross-cutting themes.

## The More Ambitious the Targets, the More Likely It Appears That Innovative Technologies Will Be Needed to Attain Them

We asked authors to reflect on the “Global Health 2035” goals, as well as on the Sustainable Development Goals (SDGs) and other relevant disease-specific goals set out by different campaigns. In their paper on translational research for TB elimination [3], Christian Lienhardt and colleagues discuss the World Health Organization (WHO) End TB Strategy, which aims to end the global TB epidemic by 2035. In their paper on ending AIDS [4], Glenda Gray and colleagues discuss the global health community’s goal of “an AIDS-free generation” [5]. The SDG for health, SDG 3, calls for an end to newborn and child deaths within 15 years. But such bold “zero” targets will remain in the realm of fantasy without a massive, concerted, and global R&D effort. For example, as the authors in this collection acknowledge, ending AIDS, TB, and malaria will require efficacious preventive vaccines against these three diseases, ever-improving medicines, and rapid point-of-care diagnostics [3,4,6]. We know of no credible empirical research showing that the ambitious zero targets for global health can be reached with today’s technologies alone.

## Innovation Must Go beyond Technologies

A striking theme that cuts across this collection is that technological innovation alone will have no impact unless it is accompanied by innovations across the entire spectrum, from basic science all the way through to health systems and financing. As Peter Hotez and colleagues noted in their article on eliminating NTDs, “We have learned that new tools will not deliver themselves” [7]. Delivery systems worldwide need to be improved, they say, which requires “a different type of innovation that is dependent on local capacity and implementation science, where we move from the question of ‘can this work,’ to ‘how can it work here?’” In their paper on the science of scale-up, Kruk et al. make the case for major investment in what they call “policy and implementation research” [8], which can be defined as the “systematic and rigorous analysis of which delivery approaches worked across a variety of health needs and which did not” [9]. And Cyril Engmann and colleagues, in their examination of “promising and important innovations” that could have “transformative potential on the survival and wellbeing of mothers and children,” highlight important innovations in improving access to treatment, financing of maternal and child health services, and the measurement of progress [10].

The smallpox eradication campaign, which is one of the greatest success stories in global health, provides an object lesson in the importance of multiple types of innovation. Eradication was made possible only through a series of innovations after Edward Jenner’s original breakthrough in discovering the vaccine:

- Product innovation: The development of the heat-stable, freeze-dried vaccine by Leslie Collier at the Lister Institute of Preventive Medicine in the United Kingdom dramatically improved the vaccination “take rate” in tropical settings. Prior to this innovation, tropical countries were forced to use cumbersome methods to try to distribute liquid vaccine—in Peru, for example, liquid vaccine was taken into the field in kerosene refrigerators mounted on the back of mules [11].
- Procedure innovation: The development of the bifurcated needle by Benjamin Arnold Rubin at Wyeth Laboratories simplified vaccination procedures, reduced the quantity of vaccine used, and gave a better take rate than earlier vaccination techniques [11].
- Health policy innovation: Lack of funds forced the global smallpox campaign to mobilize not only under-used health personnel but also community teachers, religious leaders, and village elders [12].
- Strategy innovation: William Foege’s proposition to give priority to surveillance and containment of outbreaks (“ring vaccination”), instead of mass vaccinations, allowed for as little as 7% of a population to be vaccinated, yet still removed the disease from populations and much more quickly than mass vaccinations [13].

As Mary Moran argues in her Perspective, global health goals cannot be reached unless the two different worlds of innovation and public health can be brought together [14]. “At the moment,” she argues, “These worlds are often disconnected, with major gaps to be bridged at both the intellectual and practical levels before we can truly reach a grand convergence in health.”

## Urgent Action Is Required to Close the R&D Funding Gap

We believe that the international community is under-investing in R&D for conditions of poverty and, as a result, progress is being needlessly delayed. Only about 1%–3% of the world’s health R&D is targeted at “the big five” diseases that disproportionately affect low- and middle-income countries [1]. The estimated shortfall in annual funding is at least US$3 billion [15], a gap that could easily be closed within just a few years through increased financing from government and philanthropic donors, high-burden countries (especially middle-income countries, such as Brazil, China, and India), and the private sector. A number of middle-income countries, which Morel and colleagues have called “the innovative developing countries,” have already shown the value of domestic investment in health R&D and are forming new kinds of health innovation networks and partnerships [16].

Increased financing for such R&D will bring benefits that go beyond health, particularly economic benefits. The initial investment by the March of Dimes of about US$26 million to develop the polio vaccine prevented over 160,000 polio deaths and around 1.1 million cases of paralytic polio in the US alone—generating a net benefit of about $180 million in treatment-cost savings [17,18]. The promising innovations discussed in this *PLOS* collection, and in the recent “Innovation Countdown 2030” report (http://ic2030.org/report/), are likely to bring similarly impressive economic returns. For example, Jamison and Hecht estimate that every US$1 invested in HIV vaccine development would return between US$2 and US$67, assuming that the R&D costs are about $900 million annually and that a vaccine of 50% efficacy becomes available by 2030 [19].

As Trevor Mundel argues in his Perspective, it would be enormously valuable for the international community to develop a more strategic and data-driven approach to investing in global health R&D [20]. Such an approach would be based on considering—and linking—the most timely data on the burden of disease, the health tools that are most needed, the most promising candidates in the R&D pipeline, the costs to develop and deliver them, and the estimated health and economic returns from their deployment.

The international community also needs to do a better job of monitoring progress on global health R&D. An important debate is now underway about which indicators should be adopted to monitor progress towards the SDGs, but this debate has largely overlooked the need for indicators on health R&D and innovation. Policy Cures, an independent research and policy group, has urged the community to adopt a set of pragmatic indicators, which we fully support, that would include (i) globally collected data on public, private, and nonprofit investment in R&D and on the number of new registered health technologies related to the diseases that disproportionately affect those in low- and middle-income countries, and (ii) nationally collected data on R&D expenditure as a percentage of gross domestic product (GDP) [21].

## Conclusion

The prospect of achieving a grand convergence in global health within a generation, averting about 10 million deaths annually from 2035 onward [1], represents an unprecedented opportunity to boost human development worldwide. This opportunity can only be realized through a serious, renewed effort to step up investments in R&D to tackle the health conditions of poverty.

## Abbreviations

GDP: gross domestic product
R&D: research and development
SDG: Sustainable Development Goal
TB: tuberculosis
WHO: World Health Organization
